# Towards a Global CIs’ Cyber-Physical Security Management and Joint Coordination Approach

**DOI:** 10.1007/978-3-030-69781-5_11

**Published:** 2021-01-28

**Authors:** Vasiliki Mantzana, Eftichia Georgiou, Anna Gazi, Ilias Gkotsis, Ioannis Chasiotis, Georgios Eftychidis

**Affiliations:** 8 Norwegian Computing Center, Oslo, Norway; 9 University of Trento and Fondazione Bruno Kessler, Trento, Italy; 10 Università degli Studi di Genova, Genoa, Italy; 11 IEIIT Institute, Consiglio Nazionale delle Ricerche (CNR), Genoa, Italy; 12 SINTEF A.S., Oslo, Norway; 13 Engineering Ingegneria Informatica S.p.A., Rome, Italy; 14 Instituto Superior de Engenharia do Porto, Porto, Portugal; 15 University of Padua, Padua, Italy; Center for Security Studies (KEMEA), P. Kanellopoulou 4, 101 77 Athens, Greece

**Keywords:** Critical infrastructures, Security management, Crisis management, Physical, Cyber, Stakeholders, Communication, Coordination centre, SOC

## Abstract

Critical Infrastructures (CIs) face numerous cyber-physical threats that can affect citizens’ lives and habits, increase their feeling of insecurity, and influence the seamless services provision. During such incidents, but also in general for the security of CIs several internal and external stakeholders are involved, having different needs and requirements, trying to cooperate, respond and recover. Although CIs security management process is well analyzed in the literature there is a need to set a common ground among different CIs, thus reducing administration/coordination overhead and rendering the decision making and crisis management process more efficient. In this direction, this paper considers three different CIs (airport facilities, gas infrastructures, and hospitals); presents the current and emerging physical and cyber security related regulations and standards, operations, organisational and technical measure and; finally, through the discussion on gaps and best practices identified, proposes a global, cyber-physical security management and joint coordination approach. The proposed approach recommends among others that the adoption of a Holistic Security Operation Centre (HSOC) in each CI and a National Coordination Centre (NCC), supervising them, which will facilitate the communication and cooperation between the different CI operators and stakeholders, in case of an incident, that may have cascading effects to interconnected Infrastructures. The findings presented and the conclusions drawn are linked with three EU funded research projects (SATIE, SecureGas and SAFECARE), that aim to improve physical and cyber security of CIs in a seamless and cost-effective way.

## Introduction

The EC 114/2008 directive defines as Critical Infrastructure (CI) the assets, systems, and networks located in Member States which are essential to maintain the vital economic and social functions such as health, food, transport, energy, information systems, financial services, etc. The EC recognizes that these infrastructures must be protected from the disruption by natural disasters and man-made threats, and as such has launched the European Programme for Critical Infrastructure Protection (EPCIP). The importance of physical and cyber security in CIs has never been more explicit. CIs in general, and especially in transport, energy and health sectors are exposed to various physical threats (i.e. terrorism, technological accidents, natural disasters, etc.) and cyber-attacks which are emerging especially with the increasing use of Information Systems. Now more than ever, CIs must be vigilant in establishing safeguards against physical and cyber threats, as it is imperative to have a solid understanding of the risks, vulnerabilities, security processes and technologies available. In addition, it is of paramount importance for CIs to establish a standardized crisis management process to deal with attacks that threaten to harm the organisation and stakeholders.

The aim of this paper is to describe three different CI types (airports, gas infrastructures, and hospitals); present the current physical and cyber security related regulations and standards adopted; identify their security operations, as well as the organisational and technical measures deployed by each CI; and finally describe a common, cyber-physical crisis management process encompassing the involved stakeholders. Moreover, gaps and best practices related to security issues are analysed and a global approach for CIs’ cyber-physical security management and joint coordination is proposed. This approach recommends among others the adoption of a Holistic Security Operation Centre (HSOC), which will facilitate the communication and cooperation/coordination between internal stakeholders, and a National Coordination Centre (NCC) that will facilitate the communication/coordination between the HSOC and the external stakeholders. The NCC will also support the communication and cooperation/coordination between the different CI operators and stakeholders, in case of an incident that has cascading effects to interconnected Infrastructures. The findings and conclusions that are drawn, are linked with SATIE (H2020-GA832969) [1], SecureGas (H2020-GA833017) [2], and SAFECARE (H2020-GA787005) [3] projects, during the framework of which this research was conducted. These projects aim to provide solutions that will improve physical and cyber security in a seamless and cost-effective way and enhance threat prevention, threat detection, incident response and mitigation of impacts in airport facilities, gas and hospital infrastructures accordingly.

## Critical Infrastructures Description

In the following paragraphs, a short description of the three CI types is provided, presenting information about their services, main functions and business operations, as well as basic involved assets.

### Airport Facilities

Airports, being CIs that belong to the Transport sector, play a key role in people and goods transportation, as well as in regional, national, and international trade. Airports incorporate in their operational agenda passenger comfort, cost-efficiency, environmental protection, and policies for corporate and social responsibility. The interconnections and dependencies between the various systems and assets, combined with the presence of different actors and the complexity of airport operations, expose the entire environment to cyber and physical threats and make it vulnerable to various attacks.

Being a complex ecosystem, various assets and systems are involved to support the airport operations, such as staff, sensors, cabling and fiber infrastructure, networking, and systems that support airport operations and the exchange of information and data among them. There is a plethora of systems that support the airport operations such as i) the Air Traffic Management (ATM) systems, the flight tracking systems, etc., which support the Airside Operations, ii) the fuel management systems, the parking management systems, the lightning detection systems, etc., which support the Landside Operations, iii) the access control systems, the baggage screening systems, the video surveillance systems (CCTV), the Explosive Detection Systems (EDS), etc., which support the Safety and Security Operations, iv) the self-service check-in systems, the Passenger Name Records (PNR), etc., which support the Passenger management, v) the building control systems, the environmental management systems, etc., which support the Facility and Maintenance Operations, and finally, systems that allow the exchange of information and data sharing among the various systems. The most common systems found in this category at airports are, among others, the Airport Operational Database (AODB), the Geographic Information Display System, etc.

### Gas Infrastructure

Natural gas is a fossil energy source with a diverse range of uses and applications. It constitutes a major source of electricity generation, a powerful fuel for domestic (heating and cooking) and industrial use, a clean and cheap alternative as transportation fuel, and is also widely used for the production of hydrogen, fertilizers and other products. The Gas value chain can be divided in mainly three sectors: (a) Upstream (production) where the gas is extracted and processed; (b) Midstream (transmission) which generally includes the transport and storage. The gas is mainly shipped by means of high/medium pressure pipelines to downstream facilities, or other transportation media in case of Liquefied Natural Gas (LNG) (tankers, trucks and rails) and; (c) Downstream (distribution) which involves the final refinement and distribution of the gas including low pressure transport to the final users and sale. The Gas system is comprised of high-pressure gas transmission pipelines, gas compressor stations to maintain and regulate pressure, gas metering and distribution stations, cathodic protection systems installed to prevent corrosion of the pipeline, remote data transmission and telecommunication systems. Gas storage is also an important and critical part of the system. The distribution infrastructure enables the transportation of gas to the end users. Local distribution companies receive the gas in city gates, transfer points from transmission pipes and deliver it to individual customers. The delivery/distribution is done through an extensive network of small-diameter distribution pipelines throughout municipal and suburban areas. Natural gas end-users are residential, commercial, and industrial sectors and power-generation customers.

The complexity of the gas network, its diversity among transportation lines, the peculiarity of the areas crossed (remote or densely populated), the various production and storage facilities make it a challenging environment to cope with. Due to their distributed nature and often completely publicly known routings, the gas grids are prone to physical attacks, cyber-attacks (e.g. SCADA manipulations) and cyber-physical attacks. Moreover, as interconnections of gas elements, interfaces with other grids, automated monitoring and regulation loops are increasing, besides cascading consequence effects, the emergence of novel types of threatening behavior are also expected. As gas availability is critical to so many other CIs, disruption in distribution can lead to series of consequent failures or disruptions causing cascading disasters. The overall objective of the industry and the governments is to avoid such events and in case it occurs, to minimize the impact of such events.

### Hospitals

Health sector is responsible for delivering services that improve, maintain or restore the health of individuals and their communities; protect population against health threats as well as consequences of ill-health and; provide equitable access to people-centered care [4]. According to WHO, hospitals complement and amplify the effectiveness of many parts of the health system, providing continuous availability of services for acute and complex conditions [4]. Hospitals depending on their mission, offer different services such as pharmacy, pathology, radiology, nursing, acute (e.g. emergency department or specialist trauma centre, burn unit, surgery etc.); specialized (e.g. cardiology or coronary care unit, intensive care unit etc.) outpatient and chronic treatment etc. They are expected to provide appropriate and responsive care and; ensure acceptability and accessibility to its services.

Moreover, several new technologies, ranging from Internet of Things (IoT), wearable external and implanted medical devices (skin patches, insulin pumps and blood glucose monitors), order entry and administrative Information Systems (IS) to laboratory and operation theatre IS, have been adopted in hospitals. There is a widespread understanding of the need to balance utility and efficacy with privacy and security in innovation; however, technology is boosting more quickly than the creation, application, and adaptation/update of security measures. In addition to cyber-threats, physical threats are increasingly growing and even healthcare facilities are not immune to them. Hospitals are generally open to the public with multiple entrances, which means identification and baggage of visitors is almost never screened, leaving hospitals vulnerable to physical attacks. It has been reported that hospitals are twice more likely to experience a physical attack incident than a cyber-attack or breach [5].

## Security and Protection of Critical Infrastructures in EU

In the following paragraphs, the three different CIs’ (airport, gas infrastructures, hospital) crisis management related issues, such as regulatory framework, security operations, systems and technologies, as well as the crisis management process and engaged stakeholders are described.

### Operation and Security Regulatory Framework

The first official effort for the preparation of a strategy to protect CIs was initiated by the European Council in 2004. In 2006, EU set the parameters for the implementation of the EPCIP [6]. In 2008, the European Council Directive 2008/114/EC (evaluated on 2019 through public consultation and pending to be revised) established a procedure for the identification of and designation of European critical infrastructures (‘ECIs’), focusing on the Energy and Transport sector, and the assessment of the need to improve their protection [7]. In accordance with the Regulation (EU) 2016/679, organisations including CIs, must protect natural persons while processing personal data and exchanging of such data. The principles of the EU Directive 2016/1148 (NIS Directive) concerning “measures of a high common level of security of network and information systems across the Union” are also applicable to CIs [8].

In the context of **airports** and additionally to the aforementioned, one year after the September 11 attacks, EU adopted a set of aviation safety and security rules based on Regulation (EC) No 1592/2002 and Regulation (EC) No 2320/2002 [9, 10]. In 2008, the EU extended the safety rules in order to cover the aircraft operations and aircrew licensing and training (Regulation (EC) No 216/2008), while in 2009 the extended Regulation (EC) No 1108/2009 covered the safety aspects of aerodromes, air traffic management and air navigation services. Currently, EC regulation 300/2008 establishes common rules in the EU to protect civil aviation against acts of unlawful interference, which pertain to the screening of passengers, cabin baggage and hold baggage, the airport security (access control, surveillance), the aircraft security checks and searches, the screening of cargo, mail, airport supplies and the staff recruitment and training. In addition, a national authority for aviation security must be appointed while establishing a national civil aviation security and quality programme. The detailed measures for the implementation of the common basic standards on aviation security are updated in “Commission implementing Regulation (EC) No 2015/1998”. Moreover, the International Civil Aviation Organization (ICAO) works with Member States and industry groups to reach consensus on international civil aviation Standards and Recommended Practices (SARPs) and policies. The regulations and policies suggested by ICAO are adopted by the ICAO Member States to ensure that their local civil aviation operations and regulations conform to the suggested norms, to ensure safety and security. ICAO’s annex 17 sets the preventive security measures relating to access control, screening of aircraft passengers and their cabin baggage, screening of hold baggage, screening of cargo and mail, measures for handling special categories of passengers, for protecting Information Systems (IS) etc.

With regards to **Gas CIs**, several initiatives and regulations specifically on the Gas sector focus on security, pinpointing the need for enhancing protection of such CIs, such as the European Energy Security Strategy [12], the stress tests on the resilience of the EU gas system [13] and the EU Regulation 2017/1938 on Security of Gas Supply [14]. The Directive 2009/73/EC establishes common rules for the transmission, distribution, supply and storage of natural gas. It lays down the rules related to the organisation and functioning of the natural gas sector, access to the market, the criteria and procedures applicable to the granting of authorisations for transmission, distribution, supply and storage of natural gas and the operation of systems [15]. Regulation (EC) No 715/2009 sets non-discriminatory rules for access conditions to natural gas transmission systems considering the special characteristics of the national and regional markets that ensure the proper functioning of the internal market in gas, the access conditions to LNG facilities and storage facilities taking into account the special characteristics of the national and regional markets, and facilitating the emergence of a well-functioning and transparent wholesale market with a high level of security of supplying gas and providing mechanisms to harmonise the network access rules for cross-border exchanges in gas.

In the context of **hospitals**, there is also regulation on medical devices (Regulation (EU) 2017/745 of the European Parliament and of the Council of 5 April 2017 on medical devices) [17]; on in-vitro diagnostic medical devices (Regulation (EU) 2017/746 of the European Parliament and of the Council of 5 April 2017 on in vitro diagnostic medical devices) [18] etc.

### Security Measures and Technologies

For CIs of any sector, the selection among the security measures to be adopted depends greatly on the assets that need to be protected, their location and operational specifications, the threats, vulnerabilities, and risks associated to them. Thus, applying appropriate protection for the CI, by implementing the suitable security measures and technology, requires an understanding of the environment under consideration as well as the threats to which it is exposed.

#### Cyber Protection Measures

In order for CIs to: (a) prevent or at least reduce unauthorized access, use, disruption, deletion, corruption etc.; (b) respond effectively, timely and efficiently and; (c) minimize the impact of attacks to their network, information technology and systems, it is important to take both organisational and technical measures, as analysed below.

Organisational measures might include (a) the assessment of cyber risks, which is used to identify, estimate, and prioritize risk to organisational operations, organisational assets, individuals, other organizations, and the Nation, resulting from the operation and use of information systems [19]; (b) development and adoption of both generic and case specific laws, standards, plans and policies that outline cyber security measures and crisis management procedures, and; (c) staff training on cyber security protection and crisis management issues, standards, plans and protocols.

Technical measures adopted, include among others the following: authentication, access control (authorization), data confidentiality and integrity, backup, tracing systems, log files, communication security, firewalls, traffic monitoring systems, etc. A security by design approach should complete the aforementioned countermeasures, focusing on the cyber security aspects for new devices or systems, that need to be planned and implemented already from the beginning, meaning the procurement, design, development and maintenance phases. For securing networked devices and assets inventories should be created and maintained, as they can ensure a sound understanding of the systems and their components; support configuration and automated remediation management processes [20] and software should be regularly patched and updated.

In addition, it is also crucial to have a clear understanding of actual cyber security strategies and controls implemented at targeted infrastructures, such as: a) with regards to **airports**: Passenger Data Records, Flight Display System and Management, etc., b) with regards to **Gas CIs**: Supervisory Control and Data Acquisition (SCADA)which is one of the most common types of industrial control systems (ICS), being responsible for providing automated control and remote human management of essential commodities and services, c) with regards to **hospitals**: medical devices, e-health services etc.

#### Physical Protection Measures

Investments in CIs physical security monitoring are likely to increase (21). Even if human observers theoretically offer greatest security, it is also necessary to take account the drawbacks of human inattention and limited senses. Generally, security monitoring requires several sensor devices that are based on more or less sophisticated technologies, basically according to the application need. Examples of tools and capabilities to create safe and secure **hospital** environments for patients, staff and visitors include perimeter protection, physical barriers/bollards, guards, lighting, audio and video surveillance, access control, intelligent controllers, Intrusion Detection System (IDS), Physical Security Information Management (PSIM) systems, Unmanned Aircraft Systems (UAS), anti-drone technologies, motion and temperature sensors, CBRNE sensors. These physical security measures though are applicable also to airports, **Gas Networks** or other CIs.

**Airports** implement and adopt some additional measures and technology solutions, such as baggage screening systems, baggage handling systems, Explosive Detection Systems (EDS), passenger screening systems, standardized screening techniques, which all passengers must undergo (e.g. baggage X-rays, metal detecting scans). Despite the fact that these measures are used for physical security, cyber security measures must be adopted to protect them.

In the context of **Gas CIs** the operation and integrity of the Gas system is usually handled by SCADA system, which monitors and controls systems remotely, both for operational and safety/security purposes. Remote Terminal Units (RTU) or local controls systems on production sites, pipelines, compressor and pump stations, regulation and metering stations, are connected to the SCADA by means of available communication media.

### Security Related Operation Centers

Critical Infrastructures, based on their aim, functionalities and regulatory frameworks (as explained above), adopt several security related operations centers, given that any damage to a critical infrastructure, its destruction or disruption by natural disasters, manmade events or technological accidents, may have a significant negative impact for the security of the EU and the well-being of its citizens.

In addition to the previous and in order to deal with security issues on an organisational and technical level, **airports** have incorporated in their facilities, different operation centers to safeguard them in their daily routine. The Security Operation Centre (SOC) is a generic term describing part of or the whole platform whose purpose is to provide detection and reaction services to security incidents [21]. SOC monitors the security level of an organisation on an ongoing basis and comprises of a security team using various technological solutions in order to oversee security operations and to collect data and syslog to detect, identify, analyse, investigate and report cybersecurity incidents. SOC architecture models can differ based on airport’s needs and preferences. There are dedicated or internal SOC (team within organisation), virtual SOC (team works remotely), and co-managed SOC (internal IT collaborating with outsourcing vendor). The Emergency Operations Centre (EOC) is a facility operating to manage disaster emergencies. It is the place where information management, allocation and coordination of resources, and recovery actions take place. The Network Operations Centre (NOC) manages, controls, monitors and maintains the network functionality and operations across various platforms, media and communication channels (internal or external). The Airport Operations Centre incorporates a selection of the centers (including the previous ones) based on the operational needs of each airport. AOC constitutes an operational management structure that allows a common operational view to airport stakeholders in order to communicate, collaborate, coordinate and decide on the progress of airport operations. In case of a security incident the SOC operators would detect the security incident and immediately inform the AOC which is the focal point for information collection and sharing once an emergency is declared. Depending on the nature of the attack, the required stakeholders are determined, and the response and recovery measures are decided.

A similar structure but in a less sophisticated level applies also in **gas CIs**, where the Security Operation Centre, may include emergency and/or network operations centers. The SOC of a gas infrastructure is a secure control room within each infrastructure, where the SCADA, fire alarm systems, CCTV, and other sensors and systems are monitored and communication systems with stakeholders are established.

**Hospitals** though most of the times and due to the “open” philosophy of being built on in order to serve patients, they do have only security and network operational centers of small scale, or in some cases they do not operate such centers.

### Crisis Management Process and Stakeholders Involved

Crisis management has been defined as “the developed capability of an organisation to prepare for, anticipate, respond to and recover from crises” [22]. The full cycle of crisis management can be described in four phases (Preparedness, Response, Recovery, mitigation), with several steps following. These steps are presented in Sect. 4, linked with the proposed security coordination and operational centers of this paper.

Within these steps, several internal and external stakeholders are involved, having different needs and requirements, trying to cooperate, respond and recover from the crisis. Security stakeholders can be categorized according to their involvement and perceived proximity to the organisation into internal and external.

Based on relevant literature review and information collected from the participating CIs (airports, gas infrastructures, hospitals), the following list summarises the common internal stakeholders: Board of Directors (BoD), Data Protection Officer (DPO), Crisis Management Team, Emergency response Team, Physical security manager/personnel, IT Security manager/personnel, Technical manager/personnel, Health and Safety manager. The common external stakeholders’ category includes individuals or groups outside the organisation who can affect or can be affected by a security incident in the CI, as they are conjoint into an interdependent relationship, namely: Law Enforcement Agencies, Fire Brigade, Emergency medical services, Civil Protection, National Authorities (Prefectures, Municipalities, etc.), Ministries (e.g. Energy, Transport, Health, etc.), National Intelligence Agency, National Data Protection Authority, Interconnected/Interdependent Critical Infrastructures (e.g. power, communication, surface transportation), Computer Emergency Response Team (CERT).

In relation to **airports**, the following internal stakeholders were identified additionally to the aforementioned: Airport Duty Officer (ADO), Crisis Management Centre (CMC), Airport Operations Centre (AOC), Emergency Operations Centre (EOC)/Emergency Operations Team (EOT), Security Operations Centre (SOC)/Security Services Department, Media centre, Friends and relatives’ assistance centre. External stakeholders involved in case of crisis are: International and EU Organisations (e.g. ICAO, EASA, EUROCONTROL), Air Accident Investigation and Aviation Safety Board (AAIASB), Civil Aviation Authority (CAA)/Aviation Authority, Air Traffic Control (ATC) (e.g. ENAV), Information Security Service Providers, Telecommunication Providers, Airlines, Ground Handlers, Cargo, Concessionaires,

In the context of **Gas CI**, external stakeholders that are also informed in case of emergency are Regulatory Authorities (e.g. Energy RA).

As regards to **hospitals,** the group of external stakeholders includes additionally the Public Health Control Centres (e.g. Disease Control and Prevention) and Regional Health Authorities.

### CIs Security Management Gaps and Best Practices

Standardization of safety and security procedures has followed a sectoral approach while its maturity varies according to the criticality of the services provided. Thus, security management in the three CIs presented complies with international guidelines and standards (e.g. ICAO, ENTSO-G, WHO), which though do not offer the same level of integrity and maturity. Transfer of security organization approaches mainly from the aviation (airports) sector to the gas and to the less securitized environment of hospitals, is one of the opportunities identified in the paper to effectively address CI protection challenges. This will contribute to develop and use common management approaches and bring the security of the envisaged Cis at a comparable level.

The European Commission paved the way to integrate the management of the security of the ECIs through the EC Directive 114/2008, which was transferred to the national legislation of the member states since 2010. However, this directive addressed only the transport and energy sector and focused mainly to the threat of terrorism. Furthermore, the interconnection and interdependencies of the infrastructures have not been included at all as a systemic element of CI protection. Most of these challenges are planned to be addressed in the new relative directive, currently under elaboration.

Based on the previous analysis, reports on security management, and the daily challenges faced by the CIs, the following gaps have been identified with regards to the management of crisis and security as a whole:

- **Gap 1. Different physical-cyber security solutions implemented in different infrastructures:** Among CIs, there is a lack of uniformity in the adoption and implementation of solutions that can support and enhance crisis management processes.
- **Gap 2. De-centralised control and collection of information:** According to current usual practices, most CIs use multiple decentralised information gathering processes that run in parallel (potentially overlap). Usually, there is no single coordination point acquiring the complete set of collected data for feeding it to the interested parties.
- **Gap 3. Lack of fast communication and information dissemination:** CIs need to effectively and efficiently manage and share information (incident detection, evolution, resource allocation and management etc.), in different layers: within the CI, between the CI and its response partners, between the CI and the public, as well as among interconnected CIs.
- **Gap 4. Complexity of predicting the potential impact of an incident:** a) within the CI (i.e. fire propagation, terrorist attacks, plum dispersion, impact of toxic chemicals, radioactivity etc.), and b) among interconnected CIs, as disruptions in one sector can have cascading effects in other sectors, including cross-border.
- **Gap 5. Crisis management process understanding:** Although the crisis management process is well analyzed in literature there is a need for CIs to better understand the process, as well as to identify the involved stakeholders.
- **Gap 6. Lack of training and exercising in crisis management:** To enhance readiness and cooperation to respond to any type of complex incidents and emergencies, all involved stakeholders should perform continuous training.
- **Gap 7. Different or no security plans within each CI:** Standards and guidelines for the implementation of comprehensive plans for the security of a CI are needed at a national level (and if possible per CI sector) in order to build a common ground for all CIs. It is of high value to have a series of standardized plans (Risk and Vulnerability Assessment, Security Operations, Crisis Management, Business Continuity) related to preventive planning, day-to-day operations and business continuity management.

Despite the presented gaps, it appears that there are some best practices applied and used by the different CIs, as follows: (a) Airports and Gas CIs appear to have regulatory authorities/bodies that work with Member States and industry groups to reach consensus at international Standards and Recommended Practices (SARPs) and policies in security issues **(related to Gap 5, 6 and 7); (b)** Airports and Gas CIs use advanced physical and cyber integrated security solutions (e.g. SCADA, PLC etc.) **(related to Gap 1, 2 and 3)** and; (c) In order to deal with security issues, airports have incorporated in their structure different operation centers, e.g. AOC, SOC, in more structured and detailed way than the other two CIs **(related to Gap 2, 3, 4 and 5)**.

## Proposed Global CIs Cyber-Physical Security Management and Joint Coordination Approach

Based on the consideration of the CIs security related issues, the gaps, and best practices described in the previous paragraphs, the following recommendations are made that can enhance CIs’ crisis management process, but also security as a whole: **(a) Recommendation 1.** CIs need to develop security plans and implement integrated physical and cyber security solutions (at a minimum common level depending on their needs) to protect their critical assets across their infrastructure **(related to gap 1 and 7); (b) Recommendation 2.** CIs should integrate in their organisational structure a Holistic Security Operation Centre (HSOC) to detect, analyze, and manage cyber and physical attacks and to efficiently coordinate processes, people and technologies. Thus, a common operational picture will be achieved, and efficient information sharing will be facilitated, in order to alert operators and involved stakeholders to any potential threats or incidents **(related to gaps 2 and 3) and; (c) Recommendation 3.** The establishment of a National Coordination Centre (NCC) for CIP is also of high value. The NCC will interact with the HSOC of each CI, but also with external stakeholders (see Sect. 3.4) and other Coordination or Operational Centre (e.g. ERCC) on inter-national level. The main services and capabilities provided to the CI operators include risk and impact assessment, information gathering and sharing, and coordination of an incident **(related to gaps 4 and 6) and; (d) Recommendation 4.** A common cyber-physical crisis management process should be established and followed within each CI and at Member States level **(related to gap 5)**. In the following sections, a global, cyber-physical security management and joint coordination approach that addresses the aforementioned challenges, is presented. The proposed approach will facilitate the communication and cooperation between the different CI operators and stakeholders, in case of an incident, that may have cascading effects to interconnected Infrastructures and enhance security of CIs.

### CIs Joint Coordination Approach and Structure

A fundamental precondition for the enhancement of the security and protection of CIs is a common centre for information sharing, reporting of problems, and exchanging of good practices. To this end, the implementation of a National Coordination Centre (NCC) for CIP which interacts with a Holistic Security Operations Centre (HSOC) within each CI is crucial. As depicted in the figure below, the HSOCs will manage internally in each CI the cyber and physical security in a seamless and integrated way, while acting as the single contact point between the CI and the NCC. The NCC will be the central node of the proposed approach, fully compliant with European standards (CIWIN [23] & ERNCIP [24]), used for: i) assessing risk and map hazards and threats, assisting in this way the CI operator to assess a specific risk and gather information for setting the appropriate level of security, ii) incident reporting and information sharing with external stakeholders, such as Emergency Response Services (e.g. Police, Fire Service, Civil Protection, CERT, etc.), Public Organizations/Bodies (e.g. Ministries, Regulatory Authorities, NIS, etc.), iii) information sharing with inter-connected Infrastructures, providing the necessary information about an incident and impact propagation, in order to be prepared and avoid cascading effects, iv) exchange of information and further communication with other Coordination or Operational Centres, on one hand at national level e.g. National Civil Protection Coordination Centre, 112, etc., which in its turn would provide the necessary information to the public in a structured and organised way; and on the other hand at European or International level, e.g. the Emergency Response Coordination Centre (ERCC) in Brussels, which will contact relevant Member States and organisations, if needed**.**

**Fig. 1.**
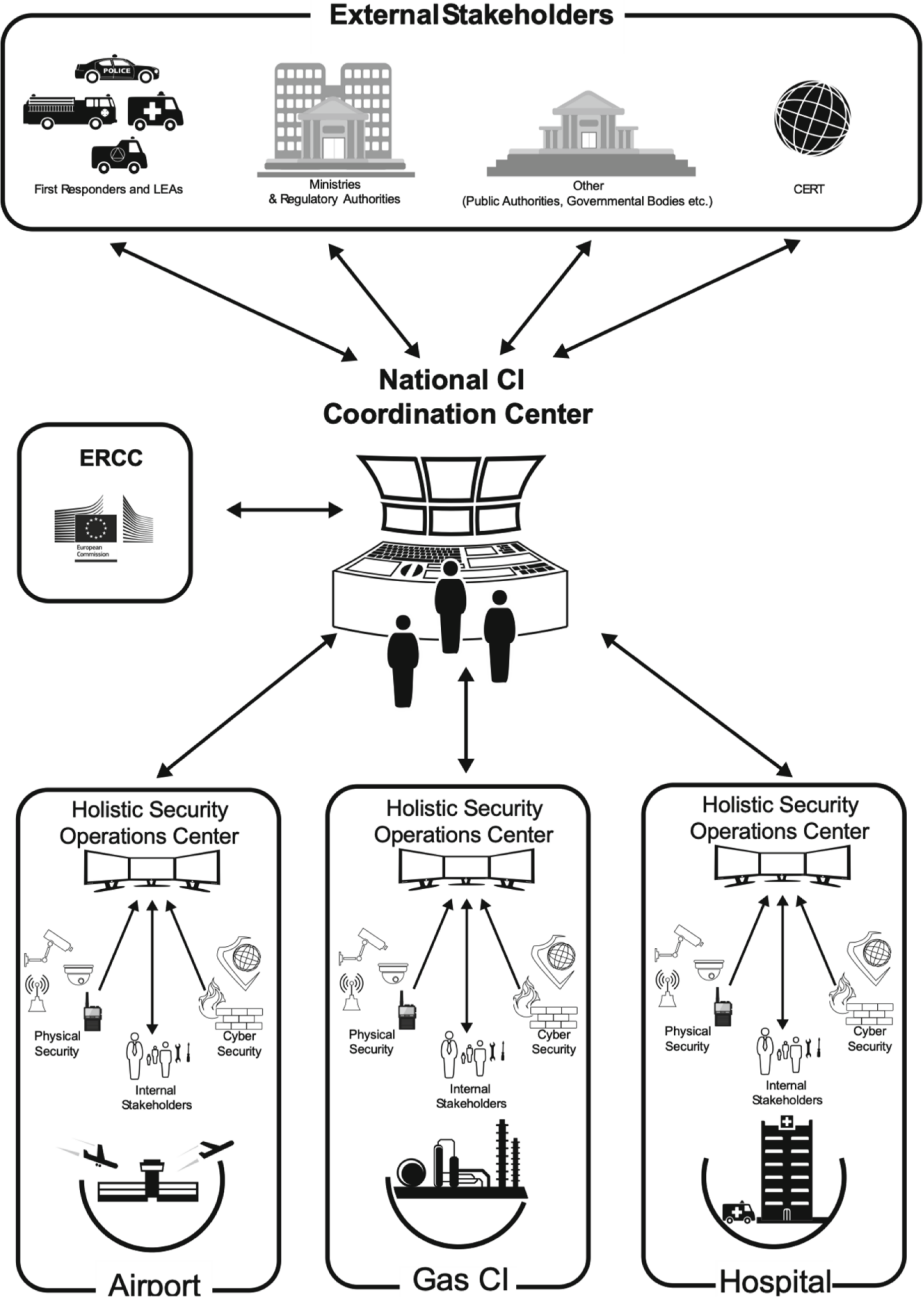
Proposed global approach for CIs cyber-physical security management

The integration of the security of CIs at the national (and at the EU level for those of European interest) is linked with the concept of interdependency and interconnection of the infrastructures. It is common practice that each infrastructure takes care of the needs to ensure their business continuity, but they are rather indifferent of the challenges the disruption of their services may cause. Their responsibility ends up with providing compensation for the related impact according to signed Service Level Agreements (SLA). However, such compensation doesn’t address the impact to the national economy and the societal disruption linked to loss of services due to interdependencies with CIs that fail to address a security incident. This is what a State Security Organization must ensure through a proper monitoring structure.

The main challenge to implement such structure is the lack of a concrete institutional and legal frame. Security pretexts and business interests are major obstructions that slow-down the required coming integration. The new European directive on CIP is a milestone ahead in this direction. The centralized role of CSIRT (Computer Security Incident Response Team) in the NIS Directive (EC 2016/1148) can be considered as the equivalent of the National Coordination node for monitoring and strengthening the physical protection of CIs at the national scale.

### Global, Cyber-Physical Crisis Management Process

Further to the above proposed approach, a cyber-physical crisis management process should be established and followed within each CI and at Member States level. This process consists of four phases (Preparedness, Response, Recovery, mitigation), with several steps linked with the proposed centers depicted in Fig. 1.

#### Preparedness:

The aim of this phase is to prepare CIs and develop general capabilities that will enable them to deliver an appropriate response in any crisis. It is a continuous cycle of planning, organizing, training, equipping, exercising, evaluating, and taking corrective actions that internal and external stakeholders should follow closely to ensure readiness. CIs is important to know which assets are vital for conducting their core activities, the potential threats against these assets, as well as their vulnerabilities. For this phase, appropriate institutional structures, supported by comprehensive policies, plans and legislation and the allocation of resources for all these capacities through regular budgets are also instrumental for thorough preparedness to crisis **(Step 1 – Develop Plans).** To improve the efficiency of the CI the appropriate tools must be in place **(Step 2 – Organise and equip).** These tools might include among others a list of contacts, hardware and software tools etc. Training and exercising are the cornerstones of preparedness which focus on readiness of all involved stakeholders to respond to any type of incidents and emergencies and on the identification of any discrepancies in terms of resources **(Step 3 – Train and exercise).**

#### Response:

Response initiates when an incident is detected by an internal or external stakeholder or the Holistic Security Operation Centre (HSOC), in a manual or automated way (e.g. monitoring networks and early-warning systems, public authorities, citizens, media, private sector, etc.) **(Step 4 – Incident Detection).** Depending on the type of the incident (cyber and/or physical) different stakeholders will collect the information needed for further investigation. Additionally, information from multiple sources, such as sensors, social media and crowdsourcing could be collected by the HSOC. The information to be gathered usually includes details relevant to the type of incident, active and passive threats, the number and the type of casualties, geospatial information, images, video, etc. **(Step 5 – Information gathering)**. The information should be collected and assessed by the Crisis Management Team (CMT) in cooperation with relevant stakeholders that identified the incident **(Step 6 – Incident assessment).** Getting a clear picture of the crisis (e.g. what happened, how many assets/people are or might be affected) is the basis for decision-making. The CMT should assess the extent of the crisis, evaluate the situation, determine, and define which response plan(s) should be activated (e.g. evacuation plan, etc.), inform the HSOC, which in its turn will communicate it to internal stakeholders and through the National Coordination Centre (NCC), external stakeholders. Based on the activated plans, response processes and procedures are executed, co-ordinated and adapted **(Step 7 – Determine plan).** It is also crucial to know the availability and current status of resources, in order to allocate them efficiently **(Step 8 – Resource Management).** HSOC is also responsible for communicating in timely and accurate manner information to internal stakeholders and to the NCC **(Steps 9 - Communication & 10 – Decision implementation).** The aforementioned steps could be repeated, until processes and assets return to business as usual or to another accepted status (demobilization) and the crisis is terminated. Demobilisation will be communicated by BoD and CMT coordinator to HSOC, which in its turn will communicate it to internal and external stakeholders through the NCC. **(Step 11 - Demobilisation).**

#### Recovery:

When crisis occurs, CIs must be able to carry on with their tasks during crisis, while simultaneously planning on how they will recover from the damage the crisis caused. Undeniably, required actions to return to normal operations and limit damage to CI and stakeholders continue after the incident or crisis. The CMT should decide the recovery actions to be taken (based on recovery plans), by cooperating closely with the HSOC, NCC, as well as internal and external stakeholders **(Step 12 – Recovery actions).** The CMT should collect and analyse evidence from the incident **(Step 13 – Collect and analyse);** and then should create an evidence report **(Step 14 – Create evidence report)**. The CMT in cooperation with its coordinator should share relative information with all internal **(Step 15 – Share relative information with internal stakeholders)** and external stakeholders (e.g. Ministries, LEAs, fire brigade, interconnected CIs). Moreover, related investigations should be assisted **(Step 16 – Share relative information with external stakeholders).** As a crisis serves as a major learning opportunity, stakeholders should review the overall process as well as plans, procedures, tools, facilities etc., and identify areas for improvement **(Step 17 – Review incidence response).** Following the evaluation, lessons learnt should be identified **(Step 18 - Debriefing)** and recommendations/revisions should be made to relevant plans, and processes **(Step 19 – Update plans)**.

#### Mitigation:

Mitigation refers to the process of reducing or eliminating future loss of life/injuries, assets and operations resulting from threats/risks through short and long-term activities. The results of the evaluation of the response actions should lead to recommendations for change, responsibilities allocation and relevant timelines in order to ensure that it will be carried out **(Step 20 – Take mitigation measures).**

## Conclusions

The work presented in this paper was based on the findings and conclusions drawn from projects implemented on three different CIs, namely SATIE, SecureGas, and SAFECARE, as well as on the normative literature. The existing operations and security regulatory framework, the security measures adopted by CIs to protect their infrastructure, the security related operations centers incorporated in the CIs’ organisational structure to safeguard them, as well as the crisis management process followed by the different CIs and the involved stakeholders, were studied. Moreover, the main gaps and potential areas of improvement of the CIs’ security management processes have been discussed. Based on the conducted analysis, a global cyber-physical security management and joint coordination approach is proposed and presented. This approach recommends, among others, the adoption of a Holistic Security Operation Centre and a National Coordination Centre, aiming to support the communication, coordination and cooperation i) between a specific CI operator and its internal and external stakeholders, and ii) among the various affected CI operators and involved stakeholders, in case of an incident that has cascading effects to interconnected Infrastructures. Further to the proposed approach, a common cyber-physical crisis management process is described and proposed to be established and to be followed by CIs, at Member States level. Following the above, the proposed CIs’ security approach aims to set common ground among stakeholders in managing incidents, thus reducing administration overhead and enhancing the process of efficient decision making and information sharing, including best practices and lessons learned. Thus, this paper offers a broader understanding of the CIs’ security management.
